# Temporary Mechanical Circulatory Support in Patients in Cardiogenic Shock Requiring Cardiac Surgery: Protected Cardiac Surgery Concept

**DOI:** 10.3390/jcm15187158

**Published:** 2026-09-15

**Authors:** Anika Nagel, Dominik Geiger, Gaik Nersesian, Daniel Lewin, Yuriy Hrytsyna, Anna Stegmann, Leonard Pitts, Julius Kaemmel, Pia Lanmueller, Volkmar Falk, Evgenij Potapov

**Affiliations:** 1 Department of Cardiothoracic and Vascular Surgery, Deutsches Herzzentrum der Charité, Augustenburger Platz 1, 13353 Berlin, Germany; 2 Charité–Universitätsmedizin Berlin, Corporate Member of Freie Universität Berlin and Humboldt-Universität zu Berlin, Charitéplatz 1, 10117 Berlin, Germany; 3 DZHK (German Center for Cardiovascular Research), Partner Site Berlin, 10785 Berlin, Germany

**Keywords:** temporary mechanical circulatory support, cardiogenic shock, protected cardiac surgery, microaxial flow pump, ECLS

## Abstract

Temporary mechanical circulatory support (tMCS) has evolved as a first-line treatment of cardiogenic shock refractory to medical therapy. In recent years, tMCS has been applied to bridge and precondition end-stage heart failure patients with structural heart disease for surgery or intervention. This review describes three clinical scenarios: preoperative stabilization and bridge-to-surgery (Type 1), planned intraoperative support in patients at high risk of perioperative low cardiac output (Type 2), and rescue support for failure to wean from cardiopulmonary bypass or postoperative heart failure (Type 3). PubMed/MEDLINE was searched for publications from January 2017 onward. It discusses microaxial flow pumps (mAFP), intra-aortic balloon pumps (IABP), veno-arterial extracorporeal life support (VA-ECLS), and combined strategies, including left ventricular unloading during VA-ECLS. Clinical applications include acute myocardial infarction-related cardiogenic shock, acute mitral regurgitation, post-infarction ventricular septal defect and advanced heart failure therapy. Current European and American recommendations support early, individualized and Heart Team-guided use in selected patients. In this review, we aim to summarize the important aspects of protected tMCS implementation in cardiac surgery and highlight some recent evidence in this field.

## 1. Introduction

Perioperative low cardiac output syndrome (LCOS) is a severe complication affecting approximately 10% of patients undergoing cardiac surgery or intervention and is associated with high mortality [1]. LCOS may also prolong ICU and hospital stay, increasing resource utilization because of a more complicated postoperative course [2]. Maintaining adequate organ perfusion is essential for favorable postoperative outcomes and prevention of multiorgan failure [3,4,5]. Temporary mechanical circulatory support (tMCS) can be used to achieve hemodynamic stabilization in patients with cardiogenic shock (CS) or perioperative low cardiac output syndrome [6].

Several tMCS devices and device combinations are currently available and can be implanted percutaneously or surgically. The most used systems include the intra-aortic balloon pump (IABP), microaxial flow pumps (mAFP) such as Impella^®^ (Abiomed, Danvers, MA, USA), the TandemHeart (LivaNova, London, UK) and (veno-arterial) extracorporeal life support (VA-ECLS) [7].

The primary aim of tMCS is myocardial recovery, although it may also serve as a bridge to heart transplantation or the implantation of a durable left ventricular assist device (LVAD) implantation [3,8,9].

**Figure 1:**
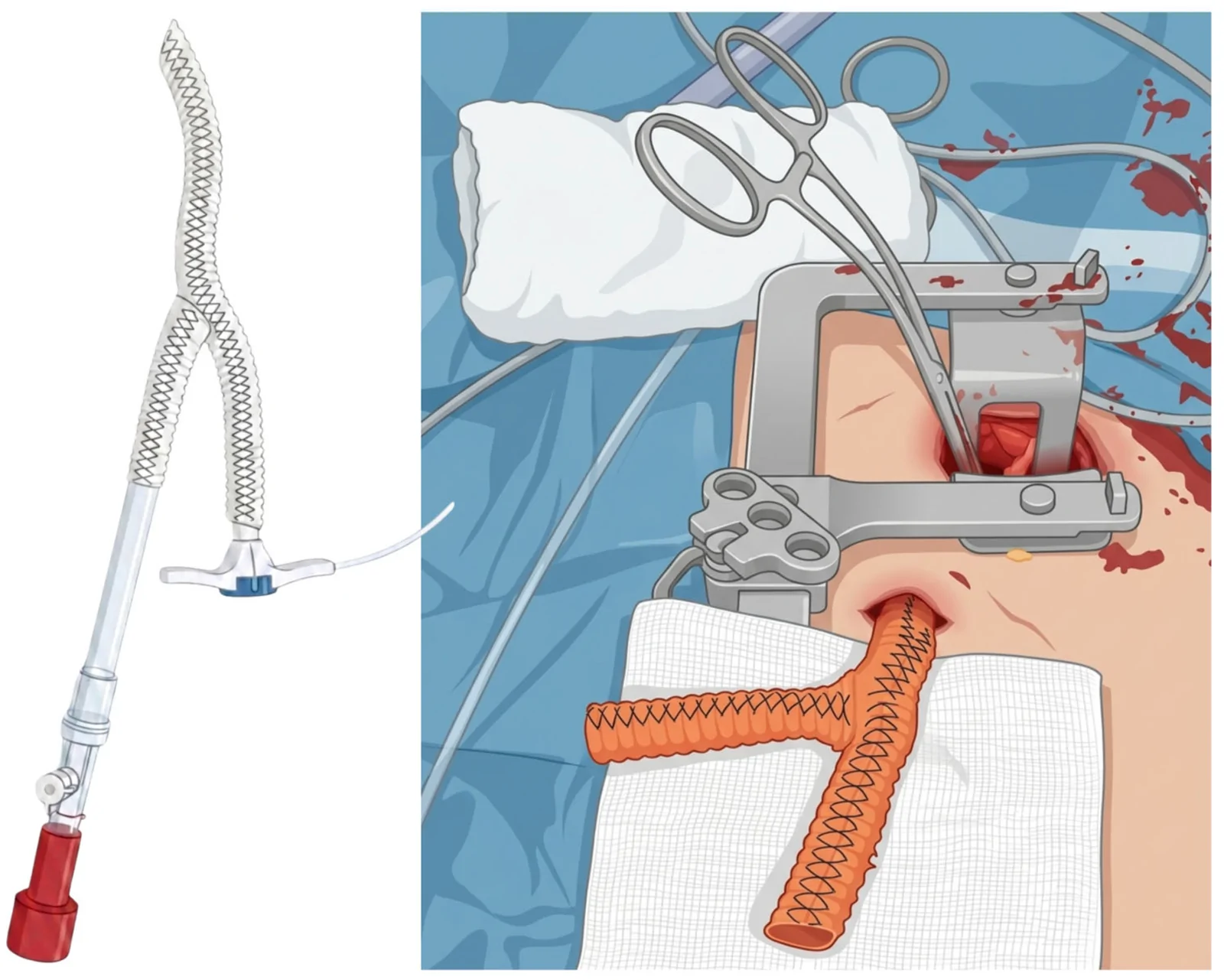
Illustration of the axillary approach as a single arterial access for VA-ECLS and mAFP implantation. The left panel shows a custom-made 10 mm vascular graft with a 15-Fr arterial cannula of VA-ECLS, and mAFP introducer placed inside the branches. The right panel shows the surgical field view with the vascular graft after end-to-side anastomosis to the axillary artery. Created in BioRender. Nersesian, G. (2026) https://BioRender.com/3d2vmeu.

mAFP devices provide flow rates from 3.5 L/min (Impella CP) up to 5.5 L/min (Impella 5.5.). Full support mAFP devices such as Impella 5.5 require surgical implantation via a vascular graft anastomosed to the axillary artery or ascending aorta [6,7]. mAFP devices directly unload the left ventricle (LV) while maintaining systemic perfusion [10,11]. The introduction of the latest generation full support mAFP has changed the management of perioperative LCOS requiring tMCS [12]. The low complication rate, the use of axillary access enabling patient mobilization and prehabilitation (Figure 1), the feasibility of implantation under local anesthesia and analgosedation, and the possibility of prolonged support have repositioned tMCS from a bailout strategy to an established treatment option [5,13,14,15].

Prospective data remain limited, and current evidence is largely based on reviews, case reports, and retrospective studies. The “EACTS/STS/AATS Guidelines on Temporary Mechanical Circulatory Support in Adult Cardiac Surgery” provide recommendations for the management of critically ill patients, including device selection, explantation, and transition to further therapy [16]. In patients with advanced heart failure or cardiogenic shock and structural heart disease, tMCS may reduce mortality and prevent perioperative LCOS [17]. In 2025, experts from the European Association for Cardio-Thoracic Surgery (EACTS) published the first consensus document on the pre-emptive use of tMCS before high-risk cardiac surgery [3].

Those guidelines emphasize early initiation of tMCS before or during surgery to prevent LCOS or limit its complications. Given the heterogeneity of patients requiring tMCS in this setting, careful patient assessment is essential, and management by an interdisciplinary heart team is strongly recommended [18].

This overview summarizes the concept of protected cardiac surgery, with a focus on temporary mechanical circulatory support in patients with coronary artery disease or structural heart disease and cardiogenic shock.

### 1.1. Concept of Protected Cardiac Surgery

A growing number of older and multimorbid patients are undergoing cardiac surgery with increasing procedural complexity. This is reflected in changing risk profiles and outcomes in national registries [19]. Cardiogenic shock and perioperative LCOS are complex syndromes with specific prognostic stages that require timely intervention. As a result, preconditioning and early stabilization strategies are increasingly used [18,20]. The concept of protected surgery emphasizes a proactive, multidisciplinary approach, with early identification of high-risk patients and planned tMCS support to prevent perioperative LCOS and improve outcomes [3,21,22]. Guidelines and consensus documents emphasize the importance of standardized indication criteria, Heart Team decision-making, treatment protocols, and structured weaning [3,23,24]. Furthermore, successful tMCS management also requires good collaboration between cardiac surgery, anesthesiology, intensive care medicine, heart failure cardiology, and perfusion services [25].

### 1.2. Types of Protected Cardiac Surgery

**Figure 2:**
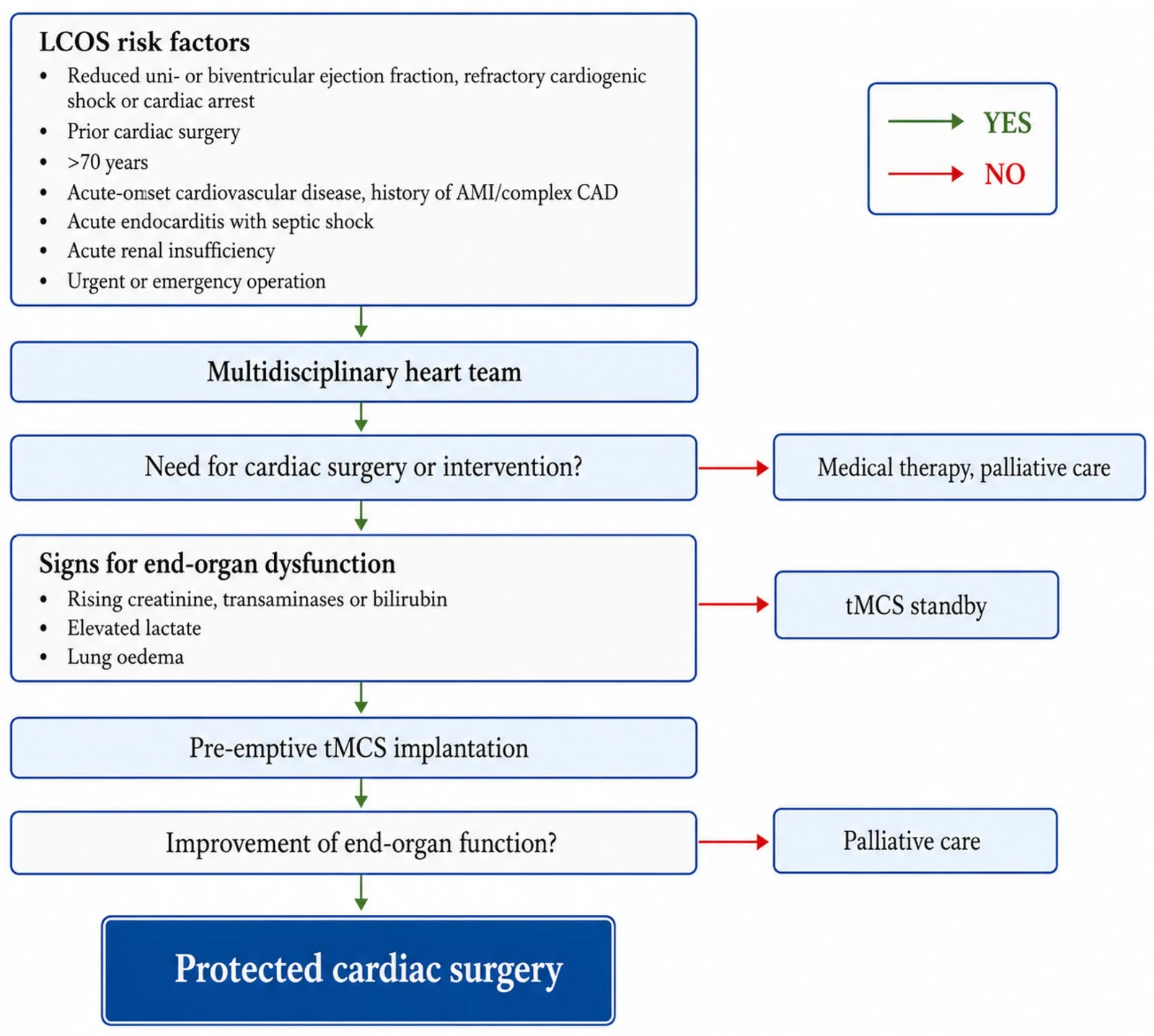
Algorithm for the evaluation of patients at high risk of periprocedural low cardiac output syndrome (LCOS) and consideration of pre-emptive temporary mechanical circulatory support [3].

The guidelines on the pre-emptive use of tMCS define three types of protected cardiac surgery based on the timing of tMCS initiation [3]. Type 1 involves preoperative optimization of patients with impaired hemodynamic, metabolic, and respiratory status. High-risk patients are identified according to predefined criteria, such as impaired ventricular function, acute kidney injury, advanced age, or a history of previous cardiac surgery, and are subsequently discussed by the Heart Team. Depending on the degree of end-organ dysfunction, pre-emptive tMCS may be indicated. Once end-organ function improves, protected cardiac surgery or intervention can be performed. Depending on the underlying cardiac pathology, different devices or device combinations are recommended [3] (Figure 2).

**Figure 3:**
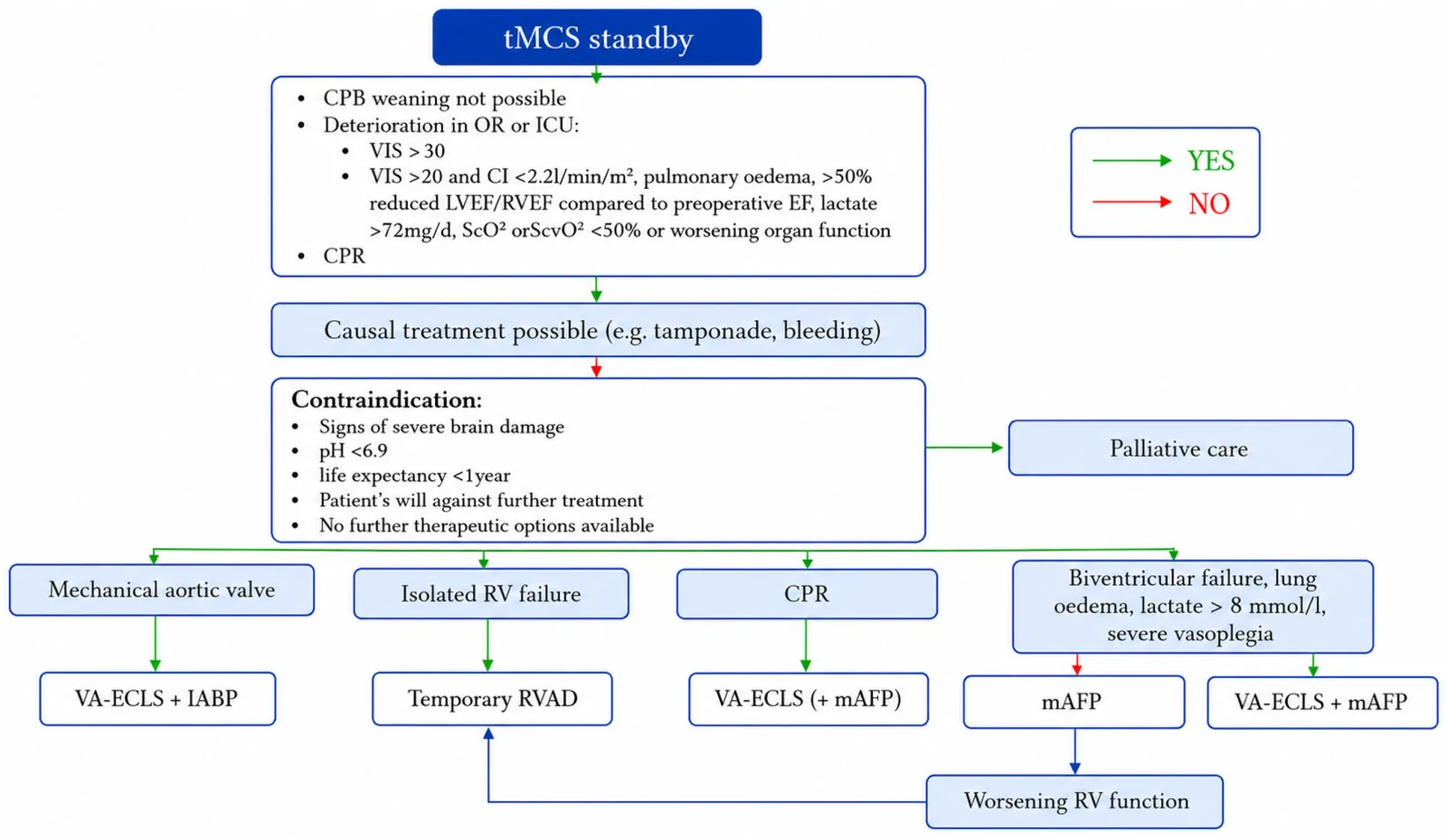
Algorithm for the intraoperative and postoperative implementation of temporary mechanical circulatory support (tMCS). The algorithm summarizes previously published recommendations and algorithms from the EACTS Expert Consensus Document on Protected Cardiac Surgery and the EACTS/STS/AATS Guidelines on Temporary Mechanical Circulatory Support in Adult Cardiac Surgery [3,16]. Abbreviations: CPB, cardiopulmonary bypass; IABP, intra-aortic balloon pump; ICU, intensive care unit; LVEF, left ventricular ejection fraction; mAFP, microaxial flow pump; MR, mitral regurgitation; OR, operating room; RVAD, right ventricular assist device; RVEF, right ventricular ejection fraction; ScvO_2_, central venous oxygen saturation; VA-ECLS, veno-arterial extracorporeal life support; VIS, vasoactive-inotropic score.

Types 2 and 3 of protected cardiac surgery involve the intraoperative application of tMCS. This may be planned in patients at high risk for LCOS (Type 2) or initiated emergently in patients developing intraoperative LCOS, including those who cannot be weaned from cardiopulmonary bypass (CPB) or develop acute left or right ventricular failure (Type 3) [3] (Figure 3). The aim of this concept is to avoid the secondary use of postoperative tMCS in hemodynamically decompensated patients (Table 1).

## 2. Methodology

The literature on temporary mechanical circulatory support in protected cardiac surgery was reviewed using PubMed/MEDLINE (https://pubmed.ncbi.nlm.nih.gov). Publications from January 2017 onward were considered, together with the earlier PROTECT I and II trials because of their relevance to the topic. The review included studies on coronary artery bypass grafting, mitral valve surgery and intervention, heart transplantation, left ventricular assist device implantation, and ventricular septal defect repair. Search terms covered cardiogenic shock, temporary mechanical circulatory support, protected cardiac surgery, Impella (Abiomed, Danvers, MA, USA), microaxial flow pump, veno-arterial extracorporeal membrane oxygenation, intra-aortic balloon pump (Getinge/Datascope Corp., Wayne, NJ, USA), and terms related to the respective procedures. Original studies, observational cohorts, case series, clinical trials, registry analyses, consensus documents, and current guidelines were included. Publications were selected according to their relevance to protected cardiac surgery and summarized narratively. No meta-analysis was performed.

## 3. Clinical Applications of Protected Cardiac Surgery

### 3.1. Bridging Patients to Surgery

Preoperative tMCS has improved outcomes in selected high-risk patients [15,30,31]. In patients with acute myocardial infarction (AMI) complicated by ventricular septal defect (VSD) or severe mitral regurgitation (MR), tMCS may serve as a bridge-to-decision or bridge-to-surgery approach by stabilizing patients before high-risk interventions [32]. This strategy may allow surgery in initially inoperable patients, avoid emergency procedures in unstable patients, and provide time for multidisciplinary Heart team evaluation and treatment planning [3,30]. Furthermore, the ESC guidelines for acute and chronic heart failure give a class IIa recommendation for tMCS use in CS to restore hemodynamic stability and allow definitive treatment [29].

Various algorithms outline device selection and timing of tMCS before, during, and after surgery [3,16]. For example, VA-ECLS may stabilize patients with severe hemodynamic decompensation before valve or septal defect surgery by restoring circulation and improving end-organ function [30]. Optimal left ventricular unloading with mAFP prior to LVAD implantation was associated with lower rates of right ventricular failure, renal dysfunction, and liver injury [31]. Based on findings from multiple studies of this kind, a structured review concluded that protected cardiac surgery combines temporary mechanical circulatory support with perioperative planning to promote recovery in high-risk cardiac surgery patients [4]. An overview of the most relevant literature is presented at the end of this chapter (Table 2).

Despite the potential advantages of (pre-emptive) tMCS in patients with structural heart disease, current evidence remains limited to observational studies. Device-related complications such as bleeding, vascular injury, and hemolysis should be considered [33].

### 3.2. Preconditioning and Surgery on tMCS

#### 3.2.1. Protected Coronary Artery Bypass Grafting

Coronary artery bypass grafting remains the treatment of choice for patients with complex coronary anatomy, such as left main coronary artery disease or multivessel disease, particularly when complete revascularization cannot be achieved via PCI or when comorbidities such as diabetes mellitus or heart failure make surgical intervention the preferred option [34]. In the STICH trial, patients with ischemic cardiomyopathy and an LVEF ≤ 35% who underwent CABG in addition to guideline-directed medical therapy showed improved long-term survival compared with medical therapy alone [35]. Compared with other revascularization strategies, surgical revascularization has been linked to a lower risk of major adverse cardiac events, particularly acute myocardial infarction after the procedure [36]. Off-pump coronary artery bypass grafting (OPCAB) avoids cardiopulmonary bypass, but its benefit in patients with severely impaired left ventricular function remains controversial [37]. In addition, OPCAB does not provide automatic postoperative tMCS support unless a tMCS device has been implanted beforehand. Recent studies suggest improved outcomes with pre-emptive tMCS [4]. Protected CABG using a full support mAFP is feasible in patients with severely reduced LVEF, providing stable perioperative hemodynamic support, allowing surgical revascularization in high-risk patients, and potentially reducing the risk of postoperative LCOS [38,39,40]. Several case series have reported favorable outcomes following perioperative implantation of full support mAFPs, with few device-related or perioperative complications [38,40,41]. Patient selection for mAFP support during protected CABG requires careful consideration of the expected hemodynamic benefits in relation to the associated procedural and device-related risks [38].

In patients with AMI-related cardiogenic shock, immediate coronary angiography and PCI of the infarct-related artery are recommended when feasible. Emergency CABG is considered when PCI is not feasible or unsuccessful, or when surgery is required for mechanical complications [26,28]. Current guidelines recommend considering pre-emptive tMCS as bridge-to-surgery support in patients with AMI-related cardiogenic shock undergoing CABG. If tMCS was not implanted preoperatively, early intraoperative tMCS should be considered in patients with LVEF < 30% who become hemodynamically unstable during CPB weaning to prevent postoperative LCOS [3]. mAFP with or without VA-ECLS is recommended in these patients, depending on the individual support requirements determined by left and right ventricular function, although VA-ECLS alone may be associated with worse outcomes after CABG [3,42]. IABP is not recommended in unselected patients with cardiogenic shock due to acute myocardial infarction due to the lack of effect (Class III) [24]. It is important to distinguish between CABG in cardiogenic shock and planned surgical revascularization in cases of reduced LVEF in order to select the appropriate tMCS devices. In patients with a preoperative LVEF < 30% and/or anticipated complex or incomplete revascularization, the EACTS/STS/AATS Guidelines on Temporary Mechanical Circulatory Support in Adult Cardiac Surgery recommend the use of either an mAFP or an IABP for perioperative hemodynamic support. VA-ECLS is reserved for patients presenting with cardiogenic shock and post-acute myocardial infarction mechanical complications [16]. However, the need for postoperative tMCS has been associated with increased postoperative morbidity and mortality compared to intraoperative or preoperative mAFP implantation [43,44].

Furthermore, it is important to determine whether an acute myocardial infarction and its possible mechanical complications are present. tMCS is recommended in patients with mechanical complications of myocardial infarction as a bridge to definitive treatment (Class IIa) [24]. tMCS has become an important add-on in the management of AMI-related cardiogenic shock, helping to stabilize hemodynamic and support early revascularization [45]. tMCS can be used either as a bridge to cardiac surgery or intervention, or postoperatively to promote myocardial recovery and preserve end-organ function [45,46]. mAFP may reduce infarct size by increasing collateral coronary blood flow, activating cardioprotective pathways, and decreasing left ventricular oxygen consumption [47]. However, Kapur et al. showed that the combination of mAFP support and a 30 min delay before percutaneous coronary intervention (PCI) did not reduce infarct size compared with PCI alone in patients with anterior ST-segment elevation myocardial infarction (STEMI) without cardiogenic shock [48]. In the IABP-SHOCK II trial, routine use of an IABP in patients with AMI complicated by cardiogenic shock did not reduce 30-day all-cause mortality compared with conservative treatment; however, the device was implanted either before or after PCI [49]. This finding was supported by a retrospective analysis that found no benefit of mAFP in terms of 30-day mortality [50]. In contrast, the DanGer Shock Trial demonstrated that the use of mAFP was associated with a lower risk of death from any cause at 180 days in patients with STEMI-related cardiogenic shock [46]. The 2025 American Association for Thoracic Surgery (AATS) Expert Consensus Document: Surgical Management of Acute Myocardial Infarction and Associated Complications assigns a Class IIa recommendation to the consideration of initial tMCS in high-risk patients undergoing PCI or CABG, particularly in the setting of cardiogenic shock. The implantation of an mAFP for left ventricular unloading prior to revascularization is even listed as a Class I recommendation [45].

Percutaneous coronary intervention is a common alternative revascularization strategy to CABG in high-risk surgical patients. Data suggest that more intensive hemodynamic support during PCI improves outcomes [51,52,53]. In the PROTECT II trial, Impella 2.5 support showed a trend toward better 90-day outcomes compared with IABP support in patients undergoing high-risk PCI [54,55]. The PROTECT III trial, which evaluated mAFP-supported (Impella 2.5 or Impella CP) high-risk PCI in patients with severely reduced LVEF, was associated with significantly lower 90-day major adverse cardiac and cerebrovascular events and bleeding rates [52,54]. Early mAFP implantation may also improve outcomes in patients presenting with CS [56]. However, in one of the most recent randomized trials, elective left ventricular support using the Impella CP during complex PCI in patients with severely impaired left ventricular function did not reduce the incidence of serious clinical events compared with standard treatment [57]. The OPTIMUM trial showed that even patients considered inoperable or at high risk may benefit from revascularization with careful patient selection and hemodynamic support. In 26.7% of PCI cases, support was provided most with mAFP for LV support [58].

Regardless of whether the procedure is CABG or PCI, a heart team approach is recommended for high-risk patients with acute coronary syndrome (Class I recommendation) [16,45].

#### 3.2.2. Preoperative Conditioning and Intraoperative Support During Mitral Valve Surgery

Chronic mitral regurgitation (MR) is one of the most common acquired valvular diseases [59]. On the other hand, acute severe mitral regurgitation can lead to pulmonary edema, reduced cardiac output, and CS [60,61]. Causes of acute MR include papillary muscle rupture as a complication of acute myocardial infarction, endocarditis, acute worsening of degenerative valve disease, and rupture of the chordae tendineae [60]. Surgical mitral valve repair or replacement remains the standard treatment for severe mitral valve disease [16]. The exact timing of surgical treatment or intervention in patients with acute severe MR has not been precisely determined and varies significantly in the literature. Emergency surgical treatment of acute MR remains a surgical challenge associated with a high risk of perioperative complications [60,62]. Preconditioning with tMCS may improve the clinical condition and allows time for Heart Team decision-making [3]. IABP support in acute MR reduces aortic impedance, lowers regurgitant volume, and improves forward output without impairing LV function [59,61]. In acute MR patients, active left ventricular unloading is explicitly recommended [16]. VA-ECLS may restore end-organ perfusion but can increase left ventricular afterload, thereby worsening MR and pulmonary congestion [16,63]. For this reason, implantation of an mAFP for active left ventricular unloading, with or without VA-ECLS, is recommended [16]. Osswald et al. reported that pre-emptive mAFP implantation before high-risk mitral valve surgery is safe and feasible [64].

Early reperfusion therapy has reduced the incidence of post-infarction mechanical complications such as papillary muscle rupture (PMR) to below 0.1%. In the rare event of such complications, the mortality remains high [45,65]. In cases of acute MR from papillary muscle rupture secondary to acute myocardial infarction resulting in cardiogenic shock, mAFP should be considered for use promptly to stabilize patients prior to intervention [45,60,66]. In cases of papillary muscle rupture, active LV unloading with mAFP, with or without VA-ECLS, should be considered [3]. In the absence of cardiogenic shock and severe pre-existing conditions, emergency mitral valve surgery is recommended for patients with PMR following an AMI [45].

During mitral valve surgery for severe functional MR in patients with severely reduced LVEF or hemodynamic instability during CPB weaning, early initiation of tMCS should be considered to prevent perioperative LCOS. When valve replacement is required, a bioprosthesis combined with adequate ventricular unloading is recommended to prevent ventricular and valve thrombosis [3]. In patients with prosthetic valve thrombosis or severe stenosis of a native mitral valve prosthesis and impaired ventricular function or cardiogenic shock, pre-emptive tMCS as bridge-to-surgery support should be considered [3]. tMCS in these patients is challenging because reduced transmitral flow may lead to prosthetic valve or left atrial thrombosis. If severe left ventricular dysfunction is present, mAFP support may be preferred over VA-ECLS to maintain transmitral flow (Class I recommendation) [16,67].

For patients with a high risk associated with mitral valve surgery, the heart team may consider transcatheter edge-to-edge repair (TEER) in select cases [45]. Evidence for tMCS in patients with severe MR undergoing interventional therapy mainly comes from case reports and small series [68,69,70,71,72]. Pre-emptive mAFP implantation provided hemodynamic stabilization, enabling successful bridging to TEER and subsequent ventilator weaning [70]. It is important to note that mAFP implantation may cause chordal rupture, potentially resulting in severe MR. Pivato et al. described successful treatment with MitraClip^®^ (Abbott Structural Heart, Abbott Laboratories, Abbott Park, IL, USA) implantation [71]. It has been shown that acute MR due to papillary muscle rupture can be treated interventionally even in the setting of cardiogenic shock when supported by tMCS [68,69]. In summary, mAFP-supported transcatheter edge-to-edge repair appears feasible in patients with cardiogenic shock and severe MR, facilitating both hemodynamic stabilization and subsequent weaning [70].

#### 3.2.3. Ventricular Septal Defect

**Figure 4:**
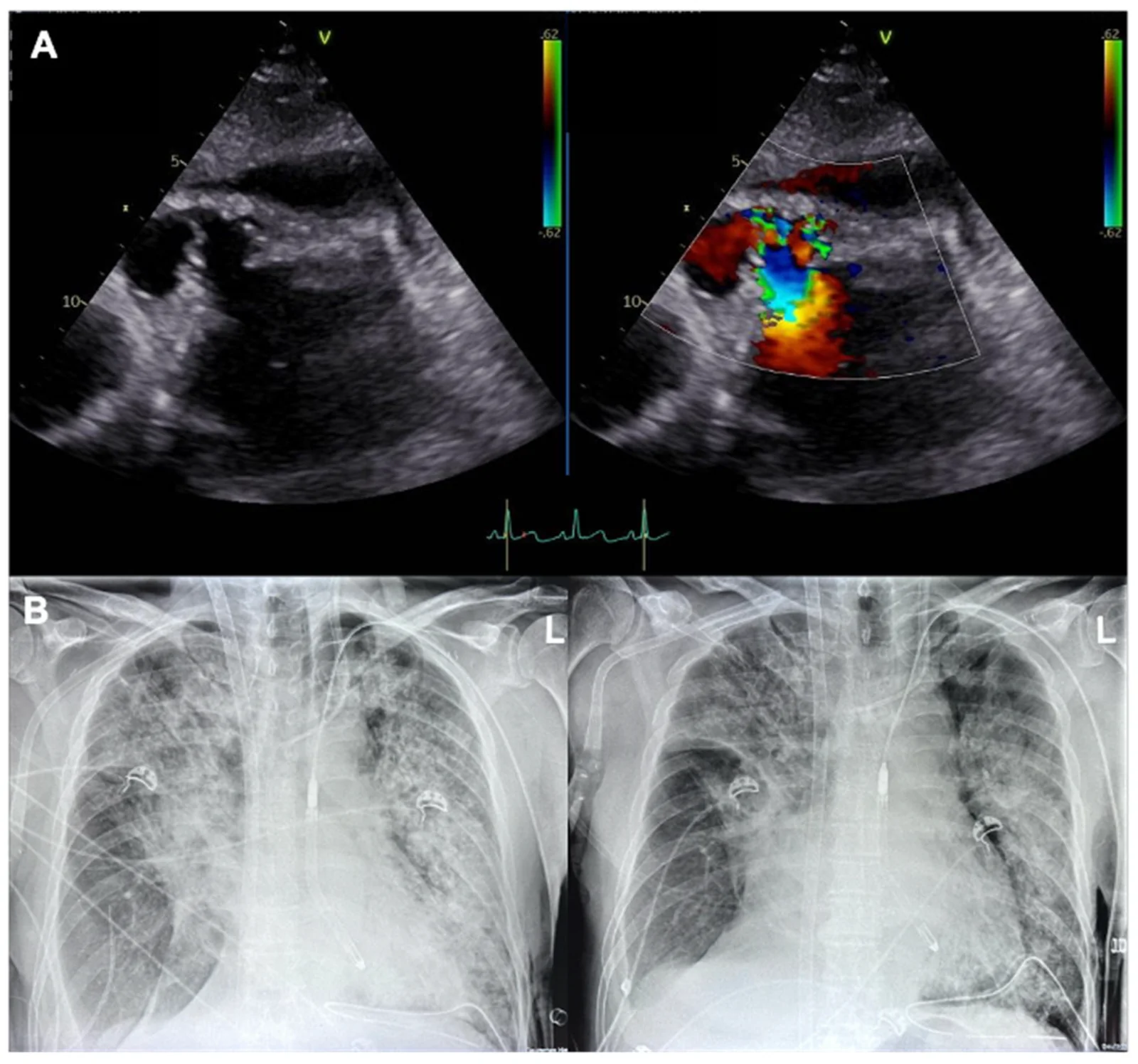
(**A**) Subxiphoid transthoracic echocardiographic view demonstrating a post-infarction ventricular septal defect with left-to-right shunting on color Doppler imaging. (**B**) Hemodynamic stabilization with a combination of Impella 5.5 with VA-ECLS (ECMELLA 2.1: single artery access with jugular venous cannulation [13]) resulted in improvement in pulmonary edema, as demonstrated on follow-up chest radiography.

Ventricular septal defect (VSD) (Figure 4) is a rare but frequently deadly complication of an acute myocardial infarction, with mortality rates exceeding 90% without surgical treatment [73,74]. Early identification and proper management are essential. The surgical treatment remains the established standard of care [73]. Despite developments in surgical techniques, the outcomes remain poor. One of the most important aspects of successful treatment is the optimal timing of the surgical procedure [74,75]. Cardiogenic shock is observed in over 80% of patients with post-infarction VSD [73]. The fundamental pathophysiological challenge in post-infarction VSD is a large left-to-right shunt that simultaneously compromises systemic perfusion while increasing pulmonary blood flow, ventricular workload, and myocardial oxygen consumption. Using a validated cardiovascular simulation model, Pahuja et al. demonstrated a shunt flow of approximately 7.5 L/min with a Qp/Qs ratio of 3.0, leading to severe pulmonary congestion and impaired end-organ perfusion. Conventional pharmacological therapies improved cardiac output and arterial pressure but unfortunately only at the cost of increased shunt flow, demonstrating their inability to sufficiently address the underlying haemodynamic disturbance [76].

Current European and American recommendations acknowledge tMCS as a bridge-to-decision or bridge-to-definitive repair strategy in patients with post-infarction ventricular septal defect, while highlighting the limited evidence supporting specific device-based approaches [45,77]. Early implementation of tMCS has been given a Class IIa recommendation in current guidelines to provide temporary circulatory support and optimize patients with post-infarction VSD prior to definitive surgical correction [45].

To choose the best suitable tMCS strategy, the size of the left-to-right shunt and the degree of ventricular dysfunction must be carefully assessed. Providing adequate systemic support while maintaining ventricular unloading, for example using VA-ECLS in combination with mAFP, IABP, or surgical LV venting [3]. Ronco et al. performed a detailed analysis of the hemodynamic effects of tMCS in VSD patients to determine the most beneficial support strategy [73].

In patients who remain hemodynamically stable, suffering only from a limited left-to-right shunt, without signs of RV decompensation or a low cardiac output syndrome, the IABP may serve as the initial mechanical support strategy. When combined with a vasodilator therapy, this approach can improve the patient’s condition and facilitate delayed surgical repair [73,77,78]. However, IABP support is insufficient in patients with right ventricular failure or severe left ventricular dysfunction [16,73]. Among the support strategies examined, mAFP support demonstrated a favorable haemodynamic profile in post-infarction VSD. Active left ventricular unloading reduced shunt and filling pressures while shifting pressure-volume loops toward lower ventricular volumes and wall stress, thereby preserving systemic perfusion [45,76,78,79]. A potential limitation is the risk of inducing a right-to-left shunt or dislodging myocardial fragments, which may result in systemic embolization [45,80]. Therefore, careful adjustment of the mAFP support is most crucial to prevent reversal of the shunt. On the other hand, according to Ruiz Duque et al., preoperative mAFP support in patients with a post-infarction VSD and a Qp/Qs ratio > 2.5 did not result in reversal of the left-to-right shunt on echocardiography [81]. In patients with a large VSD, the decision to implant a mAFP should be made with particular caution [16].

In such patients VA-ECLS is indicated. However, while VA-ECLS improved systemic circulation it simultaneously increased left ventricular afterload, leading to higher shunt flow and worsening pulmonary congestion. VA-ECLS flow should be kept as low as possible to prevent an increase in left-to-right shunting, but high enough for adequate systemic perfusion [78]. However, the 2025 EACTS/STS/AATS Guidelines on temporary mechanical circulatory support in adult cardiac surgery recommend VA-ECLS as the primary bridging strategy for patients with post-infarction VSD and cardiogenic shock before VSD closure (Class I), whereas mAFP support may be considered in selected patients (Class IIb), with serial assessment of right ventricular function to determine the need for escalation of support [16]. The ECMELLA concept combined the benefits of both systems, securing robust circulatory support while unloading the left ventricle and mitigating ECLS-associated adverse haemodynamic effects at the same time [76,77,78]. It is essential to underline, every support modality is able to substantially alter VSD-related haemodynamics. Consequently, the right selection should be individualized and guided by an experienced multidisciplinary Heart or Shock Team [77]. In contrast according to Delmas et al., mortality rates did not differ based on the tMCS strategy used, neither if mAFP alone nor in combination with IABP or VA-ECLS [82]. This shows that even if a device appears hemodynamically plausible, this does not necessarily translate into improved clinical outcomes, and current guidelines recommend VA-ECLS; however, device selection should be based on shunt size and ventricular function.

Over the past decade, tMCS has increasingly shifted the management paradigm of post-infarction VSD from emergency salvage surgery toward physiological stabilization before definitive repair [3,22]. Current guidelines advise that definitive surgical repair of post-infarction VSD carries a class I recommendation after hemodynamic stabilization is achieved [45]. The optimal timing for surgery remains a significant and controversial discussed issue. Postponing VSD repair is consistently associated with improved outcomes [74,83,84]. The reason is most likely the positive selection of patients on tMCS and the resolution of myocardial oedema, creating better condition for surgical repair. Therefore, Zbikowska et al. suggest that delayed closure may be preferable to early repair in most patients [84]. Consequently, the primary objective of tMCS is not necessarily complete haemodynamic normalization but maintenance of end-organ function, and stabilization of the patient until definitive repair can be undertaken under better conditions [3]. mAFP alone or in combination with VA-ECLS may be useful for continuous unloading of the heart and for reducing the left ventricular end-diastolic pressure (LVEDP) after repair. This may stabilize the surgical repair and allow for a safe and guided postoperative weaning process.

In summary, recent guidelines, data from physiological modelling, and emerging clinical experience strongly support a compelling rationale for preoperative unloading strategies in post-infarction VSD with considerable delay of surgery. tMCS has the potential to convert a highly unstable surgical emergency into a controlled and optimized treatment pathway. This represents one of the most compelling contemporary examples of the Protected Cardiac Surgery concept.

#### 3.2.4. Bridge to Durable Left Ventricular Assist Device

Durable left ventricular assist device implantation is one of the cornerstones of treatment for patients with advanced heart failure [29]. In recent years, tMCS has increasingly been used to bridge patients with end-stage heart failure to durable LVAD implantation [85]. Optimization of the patient’s clinical condition before surgery is associated with improved postoperative outcomes [15]. Preoperative stabilization with tMCS, particularly mAFP devices, improves hemodynamic and end-organ function and is classified as a Class IIa recommendation [3,16]. Studies suggest that patients bridged with tMCS before LVAD implantation may achieve outcomes and complication rates comparable to less severely ill patients undergoing primary LVAD implantation [86]. Although patients bridged to a durable LVAD with a partial-support mAFP demonstrated similar survival, lower rates of renal and hepatic dysfunction, higher probability for weaning from VA-ECLS, a significant reduction in mean pulmonary artery pressure and reduced catecholamine requirements were observed for those bridged with full support devices [31,87]. All things considered, escalation to full support mAFP before durable LVAD implantation was associated with improved clinical stabilization and superior 1-year survival, compared with partial support mAFP bridging [87]. In contrast, patients requiring VA-ECLS support before durable LVAD implantation have poorer survival outcomes, particularly in the presence of liver dysfunction, systemic inflammation, and obesity, all of which have been linked to increased mid-term mortality [88,89].

Several case reports suggest that preoperative mAFP implantation in patients with severely decompensated heart failure is feasible and can stabilize patients before LVAD surgery [15,88,90]. However, evidence regarding patient selection and the optimal timing of durable LVAD implantation following mAFP support remains limited. Optimization of platelet counts as well as liver and renal function should be a priority. Several risk factors for 1-year mortality after LVAD implantation have been identified, and based on these findings, Lewin et al. proposed a predictive score to support patient selection [85].

Hemolysis is a frequent complication in patients bridged to durable LVAD implantation with an mAFP and occurs more often in those supported with partial support devices. Furthermore, hemolysis has been associated with an increased risk of adverse events after LVAD implantation, including stroke, gastrointestinal bleeding, and pump thrombosis [91]. In patients bridged to durable LVAD support with a mAFP, neither the device type, duration of support, nor the use of ECMELLA at the time of implantation appeared to influence the incidence of ischemic stroke. However, both the type of mAFP device and the need for concomitant ECMELLA support at LVAD implantation were linked to a higher rate of haemorrhagic stroke during follow-up [92].

A controversial topic in the literature is whether the implantation of an mAFP increases the risk of the development or progression of aortic regurgitation due to the transvalvular path of the mAFP [93]. In patients bridged with a mAFP, the aortic valve should be carefully evaluated at the time of device removal, and an intervention should be considered in cases of significant aortic regurgitation [94]. However, unlike in smaller case series, propensity score matching by Lewin et al. showed that mAFP did not increase the risk of aortic valve insufficiency following implantation of a durable LVAD [95].

LVAD implantation can also be performed with tMCS alone and may be useful in selected patients with contraindications to CPB or elevated risk for bleeding [3,96]. One example of postoperative tMCS use after LVAD implantation is right ventricular (RV) failure. In patients who develop severe RV dysfunction following LVAD implantation that cannot be adequately managed with medical therapy or LVAD speed optimization, tMCS support may be required to maintain hemodynamic stability and end-organ perfusion [97]. Approximately one in twenty patients who have undergone LVAD implantation requires either a temporary right ventricular assist device (RVAD) or VA-ECLS due to severe right ventricular dysfunction [98]. According to the Heart Failure Association (HFA) of the ESC consensus statement, the most used tMCS systems for the treatment of severe right ventricular failure include the CentriMag RVAD (Abbott, Chicago, IL, USA), the ProtekDuo cannula (LivaNova, London, UK), VA-ECLS and the Impella RP^®^ [97]. In the cohort studied by Beller et al., VA-ECLS was associated with significantly higher mortality, as well as increased rates of vascular injury and infectious complications, compared with temporary RVAD support [98]. Therefore, as a Class 2a recommendation, early temporary RVAD should be considered in patients with severe RV failure following LVAD implantation, prior to VA-ECLS [16]. In patients with pre-existing right ventricular dysfunction who develop signs of right heart failure after durable LVAD implantation, temporary right ventricular mechanical circulatory support should be considered [3].

#### 3.2.5. Bridge to Heart Transplantation

The ongoing shortage of donor organs and increasing waiting-list times have substantially expanded the role of tMCS in patients with advanced heart failure who are waiting for heart transplantation. Patients listed for transplantation often experience worsening haemodynamic conditions despite optimal medical therapy. They may develop progressive end-organ dysfunction, recurrent ventricular arrhythmias, pulmonary congestion, or even cardiogenic shock. In these situations, tMCS plays a crucial role by stabilizing the circulation immediately, ensuring organs receive adequate oxygenated blood flow, while maintaining patients' eligibility for a transplant until a suitable donor organ is available [99,100,101]. Recent data from the ISHLT registry indicate that tMCS is now a more frequently used bridge-to-transplant depending on local allocation criteria, representing a substantial proportion of adult heart transplant recipients worldwide [102,103].

Beyond its traditional role as a bridge-to-transplant strategy, tMCS is increasingly used within a bridge-to-candidacy approach. Patients initially considered unsuitable for transplantation due to severe renal dysfunction, hepatic impairment, elevated pulmonary vascular resistance, or ongoing CS may become eligible for transplantation following haemodynamic stabilization [100]. By restoring cardiac output and reducing ventricular filling pressures, tMCS devices can facilitate recovery of end-organ function, improve metabolic status, and allow reassessment of transplant candidacy. This concept has become increasingly relevant as modern transplant programs emphasize optimization of potentially reversible risk factors before transplantation [99].

Changes in organ allocation systems have significantly boosted the use of tMCS. After the 2018 United Network for Organ Sharing (UNOS) allocation reform, the percentage of transplant candidates bridged with temporary mechanical circulatory support increased from 7.4% to 22.4%, reflecting a nearly threefold increase in tMCS utilization within modern transplant practice [99]. Notably, after the 2018 UNOS allocation reform, IABP has become the most frequently used tMCS device for bridging selected cardiogenic shock patients to heart transplantation, with its use increasing from 13.2% in the 5 years before the allocation criteria change to 29.5% in the 5 years after the change. Over the same period, VA-ECLS use increased from 4.6% to 8.4% and percutaneous left ventricular assist device use, mainly mAFP, from 2.1% to 6.2% [104]. This shift demonstrates the transition of tMCS from being a rescue therapy to an essential component of transplant procedures today. Furthermore, the marked increase in tMCS utilization after the allocation reform is also linked to reduced waitlist mortality and an increased likelihood of successful transplantation [99,100,105].

The selection of the device must be individualized according to the patient’s haemodynamic profile, ventricular function, and degree of end-organ dysfunction [23]. In the literature, intra-aortic balloon pump support is still considered an important option for selected patients with less severe hemodynamic compromise. However, in patients with heart failure–related cardiogenic shock, routine early IABP support in addition to standard care was not associated with improved 60-day survival or successful bridging to heart replacement therapy [106]. In contrast mAFP provides active ventricular unloading and substantially better haemodynamic support [107]. Several studies have reported positive outcomes with mAFP support as a bridge-to-transplant strategy [101,108,109]. Patients suffering from severe cardiogenic shock, failure of both ventricles, or simultaneous respiratory insufficiency often require VA-ECLS [21,23]. Over the past ten years, there has been a notable rise in the use of all temporary support methods. Especially VA-ECLS has shown one of the most significant increases in utilization [99]. This trend is most likely driven both by the new allocation policies and by the ability of VA-ECLS to provide rapid full cardiopulmonary support in critically ill patients. However, several studies have shown transplantation following VA-ECLS support remains associated with higher post-transplant mortality. Particularly among patients with severe end-organ dysfunction or complex underlying cardiac disease. The complication rates increase with longer tMCS support, and these devices are therefore not intended for permanent support [99,110]. For patients bridged with VA-ECLS, preoperative unloading of the left ventricle was associated with superior survival rates one year after transplantation [111].

Particularly noteworthy is the growing role of support strategies which allow mobilization [101]. Devices such as full support mAFP allow patient mobilization when implanted via the axillary artery. This allows the transplant candidate to participate in structured rehabilitation programs, and preservation of skeletal muscle mass during prolonged waiting periods. This strategy enables prolonged support durations while minimizing the risks and limitations associated with femoral cannulation [99,108,112]. The importance of maintaining best possible physical conditions is increasingly recognized as an important determinant of transplant outcomes. Frailty and prolonged immobilization correlate with increased postoperative morbidity and mortality [113,114,115]. Consequently, tMCS concepts that promote mobility provide advantages beyond haemodynamic stabilization and may enhance overall transplant readiness. In this context, the concepts of mobility on tMCS have gained popularity, emphasizing preservation of both haemodynamic stability and functional capacity while awaiting transplantation [101,108].

In addition to haemodynamic factors, tMCS may also influence transplant immunology. Recent evidence suggests that approximately one-fifth of patients supported with tMCS develop de novo allosensitization while awaiting heart transplantation. In a single-center study including 41 transplant candidates bridged with tMCS, de novo anti-HLA antibodies occurred in 22.0% of patients. The highest rate of sensitization was observed in patients supported with VA-ECLS, affecting 44.4% of this subgroup [116]. The development of allosensitization was linked to increased blood product transfusions and later durable LVAD implantation. Sensitization may prolong waiting times, complicate donor compatibility, and increase the risk of post-transplant immunological complications. These findings highlight an additional factor that should be considered when selecting bridge-to-transplant support strategies [100,116].

As waiting-list times continue to increase and transplant candidates become increasingly complex, particularly in Europe, the role of tMCS in the transplant process is expected to become more significant. Future developments will focus on earlier implementation before irreversible end-organ injury occurs, broader adoption of ambulatory support concepts, and an adaptive device selection process guided by haemodynamic phenotyping. Advances in patient selection, earlier device implantation, improved management of device-related complications, and greater experience within multidisciplinary advanced heart failure teams have led to improved outcomes [99,100,101,110,117].

**Table 2:** Publications from 2017 onward were grouped into three predefined evidence tiers: (1) guidelines and consensus documents; (2) direct clinical evidence in protected or perioperative cardiac surgery; and (3) indirect contextual evidence from cardiogenic shock, acute myocardial infarction, or non-surgical mechanical circulatory support populations. Within each tier, studies were ordered according to study design, direct clinical relevance, cohort and multicentre scope, and clinically relevant outcomes. Types 1/2/3 = preoperative/bridge, planned intraoperative, unplanned early intraoperative (rescue).

Rank	Paper	Design/Cohort	Relevant Findings
**1**	Lorusso et al., EACTS Expert Consensus on Protected Cardiac Surgery 2025 [3]	International EACTS expert consensus; no original patient cohort	Defines Type 1 (preoperative stabilization/bridge-to-surgery), Type 2 (planned intraoperative support), and Type 3 (unplanned early intraoperative rescue support).
**2**	Potapov et al., EACTS/STS/AATS Guidelines 2025 [16]	Multisociety guideline on temporary MCS in adult cardiac surgery	Indication and device selection should integrate shock stage, LV/RV physiology, end-organ function, operative complexity, access risk, and the exit strategy.
**3**	Lorusso et al., 2020 EACTS/ELSO/STS/AATS expert consensus on postcardiotomy extracorporeal life support in adult patients 2021 [21]	Multisociety consensus on VA-ECLS after cardiac surgery	Addresses patient selection, timing, cannulation, LV unloading, anticoagulation, neurological monitoring, weaning, and goals of care.
**4**	Køber et al., 2026 ESC Guidelines for the management of heart failure [24]	ESC guideline, endorsed by the EACTS; recommendation on tMCS in cardiogenic shock	Refines the classification of heart failure based on left ventricular ejection fraction, introduce a stage-based approach, and provide recommendations for mechanical circulatory support in patients with advanced heart failure.
**5**	Sánchez Vega et al., Cardiol J 2022—Surgical timing in VSD [74]	Multicentre retrospective cohort; *n* = 141 post-infarction VSD; conservative versus surgical treatment; MCS recorded	Among surgically treated patients, in-hospital mortality was lower when repair was performed from day 4 onward than within the first 24 h (37.4% vs. 65.5%; OR 0.3); in-hospital mortality was 52.3% after surgery versus 91.5% with conservative treatment.
**6**	Delmas et al., ASAIO J 2023—Impella in post-infarction VSD [82]	European multicentre retrospective registry; 17 centres participated, 28 patients were included; all in cardiogenic shock	Major bleeding 50%; in-hospital and 1-year mortality 75%. Only surgically treated patients survived; in-hospital survival was 70% in this subgroup.
**7**	Gemelli et al., Interdisip Cardiovasc Thorac Surg 2024—Impella in VSD [78]	Systematic review; 20 publications, 68 patients; 95.6% in cardiogenic shock	Surgical or percutaneous repair was performed in 72%; in-hospital mortality was 47%; 29 Impella-related complications were reported.
**8**	Singh et al., Ann Thorac Surg 2024—MCS during surgical revascularization [44]	Retrospective single-centre cohort; *n* = 378 isolated CABG procedures with LVEF < 35%; pre- and postoperative MCS groups	Overall operative mortality 3.4%; the postoperative MCS group had higher mortality and worse late survival; observational evidence.
**9**	Benke et al., J Clin Med 2022—Preventive Impella [40]	Retrospective single-centre series; *n* = 14, LVEF ≤ 30%; perioperative Impella 5.0/5.5	30-day survival 92.9%; median support duration 4 days; acute kidney failure occurred in four patients.
**10**	Osswald et al., Front Cardiovasc Med 2023—Impella in mitral valve surgery [64]	Retrospective single-centre series; *n* = 11 high-risk mitral valve operations in advanced heart failure	10/11 patients were successfully weaned; 30-day and discharge survival were both 90.9%. AKI requiring temporary dialysis occurred in 3/11 patients (27.3%), and wound infection in 2/11 patients (18.2%)
**11**	Nersesian et al., Artificial Organs 2025—Protected CABG with mAFP [38]	Retrospective single-centre Charité series; *n* = 12 scheduled protected CABG procedures with LVEF ≤ 25%; median LVEF 18%.	Seven patients achieved myocardial recovery, four received a durable LVAD, and one died on mAFP support. Overall, 2/12 patients died during the 93-day follow-up. Four thromboembolic strokes occurred in three patients during device explantation/exchange.
**12**	Mariani et al., JAHA 2023—PELS-1 [118]	International multicentre observational study; *n* = 2058, 34 centres; postcardiotomy VA-ECLS	In-hospital mortality 60.5%; among hospital survivors, 1-/2-/5-/10-year survival was 89.5%/85.4%/76.4%/65.9%.
**13**	Mariani et al., J Thorac Cardiovasc Surg 2023—ECMO timing [119]	Multicentre retrospective observational study; *n* = 2003; 1287 intraoperative, 716 outside the operating room	Postoperative initiation was associated with more complications and higher in-hospital mortality (64.5% vs. 57.5%); post-discharge long-term survival was similar.
**14**	Sommer et al., JTCVS Open 2023—Impella timing after CABG [43]	Retrospective cohort; *n* = 42 post-CABG Impella 5.0/5.5 cases; 27 simultaneous, 15 delayed	30-day survival 75.6% vs. 47.6%; 1-year survival 69.4% vs. 29.8% with simultaneous versus delayed implantation.
**15**	Møller et al., NEJM 2024—DanGer Shock [46]	Open-label multicentre RCT; 360 randomized, 355 analysed; STEMI-associated CS	180-day mortality 45.8% vs. 58.5%; HR 0.74; absolute difference −12.7 percentage points. Composite safety endpoint 24.0% vs. 6.2% (severe bleeding, limb ischaemia, haemolysis, device failure, or worsening aortic regurgitation).
**16**	Udesen et al., Am Heart J 2019—DanGer Shock design [120]	Rationale and design of the open-label RCT; target enrolment 360 patients	Defines an early Impella CP strategy plus standard care versus standard care alone, with 180-day mortality as the primary endpoint.
**17**	Zweck et al., Circulation 2024—DanGer Shock: renal endpoints [121]	Secondary analysis of the DanGer Shock RCT; AKI/RRT endpoints	AKI 61% vs. 45%, RRT 42% vs. 27%; higher pump performance and suction events were associated with AKI.
**18**	Udesen et al., JAMA Cardiol. 2025—DanGer Shock: haemodynamics/metabolism [122]	Secondary RCT analysis with invasive haemodynamic and metabolic assessment	mAFP reduced early vasoactive/inotropic requirements while maintaining haemodynamic stability and accelerated lactate normalization.
**19**	Møller et al., NEJM 2025—DanGer Shock long-term follow-up [123]	Long-term follow-up of the DanGer Shock RCT	Mortality 52.5% vs. 68.8% (HR 0.70); approximately 600 additional days alive over follow-up of up to 10 years
**20**	Morici et al., JACC 2025—ALTSHOCK-2 [106]	Open-label multicentre RCT; 101 randomized (53 IABP, 48 standard care), 7 centres; HF-CS SCAI B–D	60-day survival or bridge-to-HRT: 81% vs. 75%; HR 0.72 (95% CI 0.31–1.68; *p* = 0.45). Terminated early for futility.
**21**	Kapur et al., JACC 2026—STEMI-DTU [48]	Open-label multicentre RCT; 527 patients at 55 centres; anterior STEMI without shock; TV-mAFP plus delayed PCI versus immediate PCI	Infarct size/LV mass 30.8% vs. 31.9%; difference −1.1 percentage points (95% CI −4.2 to 2.0; *p* = 0.50). Major bleeding or vascular complications 34% vs. 6%; 1-year mortality 3.6% vs. 5.1% (not significant).
**23**	Chen et al., J Thorac Cardiovasc Surg 2017—ECLS postcardiotomy shock [124]	Observational, longitudinal, propensity score-matched cohort study of 1141 postcardiotomy patients requiring ECLS	ECLS use after cardiac surgery was associated with higher in-hospital mortality (61.7% vs. 6.8%) and increased mortality and readmission during the first year, no significant differences beyond 1 year.

Abbreviations: RCT = randomized controlled trial; MCS = mechanical circulatory support; CS = cardiogenic shock; CABG = coronary artery bypass grafting; VSD = ventricular septal defect; LVAD = left ventricular assist device; mAFP = microaxial flow pump; PCCF = postcardiotomy cardiac failure; RRT = renal replacement therapy; HRT = heart-replacement therapy; VA-ECLS = veno-arterial extracorporeal life support; OR = odds ratio; AKI = acute kidney injury; CI = confidence interval; MAP = mean arterial pressure; VIS = vasoactive-inotropic score.

## 4. Discussion

Protected cardiac surgery has become an important perioperative strategy for patients with advanced heart failure, cardiogenic shock, or a high risk of perioperative low cardiac output syndrome. The concept focuses on early, pathophysiology-guided tMCS to prevent hemodynamic deterioration and end-organ damage [3,17,125,126,127,128]. In conditions such as post-infarction mechanical complications, acute severe mitral regurgitation, or advanced heart failure requiring durable LVAD implantation or heart transplantation, pre-emptive tMCS may stabilize circulation, reduce catecholamine requirements, and improve end-organ function before surgery [3,90,129,130].

Another important aspect of protected cardiac surgery is prevention of left ventricular distension and loading during VA-ECLS support. Although VA-ECLS restores systemic perfusion, it can increase LV afterload and promote ventricular distension, pulmonary congestion, intracardiac stasis, and impaired myocardial recovery [131]. Recent meta-analyses suggest that LV unloading during VA-ECLS may reduce mortality and improve weaning success, although this benefit may come at the cost of higher complication rates. Despite this, LV unloading is a physiologically important component of protected cardiac surgery, with the optimal timing, patient selection, and device strategy remaining an important aspect that must be tailored in accordance with patients’ clinical presentation [131,132,133].

The DanGer Shock trial demonstrated that routine mAFP implantation reduced mortality in patients with STEMI complicated by cardiogenic shock, although complication rates remained high. Despite reduced vasoactive and inotropic requirements, higher rates of acute kidney injury and renal replacement therapy were observed [120,121,122]. In total, 84% of devices were implanted before PCI, even though the trial was not designed to evaluate prophylactic implantation [46]. The study also did not assess the impact of vascular access strategy, although the high rate of vascular complications suggests that large-bore femoral access may have contributed to adverse events. Despite these potential benefits, tMCS remains associated with significant complications. Major concerns include bleeding, thrombosis, systemic embolization, vascular injury, limb ischemia, and access-site complications, particularly in perioperative patients requiring anticoagulation [6,7,134,135,136]. Anticoagulation should consider thrombotic risk, bleeding risk, and the requirements of the specific device. After tMCS initiation, anticoagulation with unfractionated heparin or a direct thrombin inhibitor is recommended (Class I). In patients with bleeding, anticoagulation may be reduced or stopped depending on the severity and site of bleeding, bleeding control, tMCS flow, and thrombotic risk [16]. Prolonged support may also lead to hemolysis, infection, renal dysfunction, and limited mobilization [6,25,70]. Patient-centered cardiac surgery requires clear communication of the potential benefits and risks of tMCS, allowing patients to participate in decision-making whenever clinically feasible.

Historically, fewer than 50% of adult patients receiving ECLS for treating postcardiotomy cardiogenic shock survive to hospital discharge [124]. However, CS itself carries mortality rates of up to 75%, particularly in patients with SCAI stage E shock [137,138]. Increasing evidence suggests that earlier tMCS initiation may improve outcomes, particularly in AMI-related CS [25,139,140,141,142,143]. Implantation before revascularization has been associated with improved survival [16,143]. Outcomes also differ depending on the underlying etiology of shock. While AMI-related CS has been extensively studied, evidence in CS caused by decompensated chronic heart failure remains limited [142].

Even in postcardiotomy LCOS, where prognosis is poor, rapid tMCS implantation has been associated with higher survival rates [16,118,119,144,145]. Although current risk scores remain imperfect, most highlight the importance of early implantation and active LV unloading [144,145]. Dedicated cardiogenic shock teams, institutional MCS protocols, and treatment at high-volume centers have also been linked to improved survival [25,137,144,146,147]. Although early tMCS implantation is increasingly advocated, the optimal patient selection, timing, and device strategy remain uncertain. Consequently, broader adoption should be accompanied by careful risk–benefit assessment and treatment within experienced multidisciplinary centers.

Patient-centered outcomes and psychological well-being should also be considered, as depression and suicidality are relevant concerns in patients undergoing advanced cardiac therapies, particularly in those receiving LVAD support [148]. Postoperative delirium is also common after cardiac surgery and is particularly relevant in patients requiring temporary mechanical circulatory support, with reported rates above 50% in patients receiving VA-ECLS. It may complicate the clinical course and should therefore be prevented and treated with appropriate measures [149].

**Figure 5:**
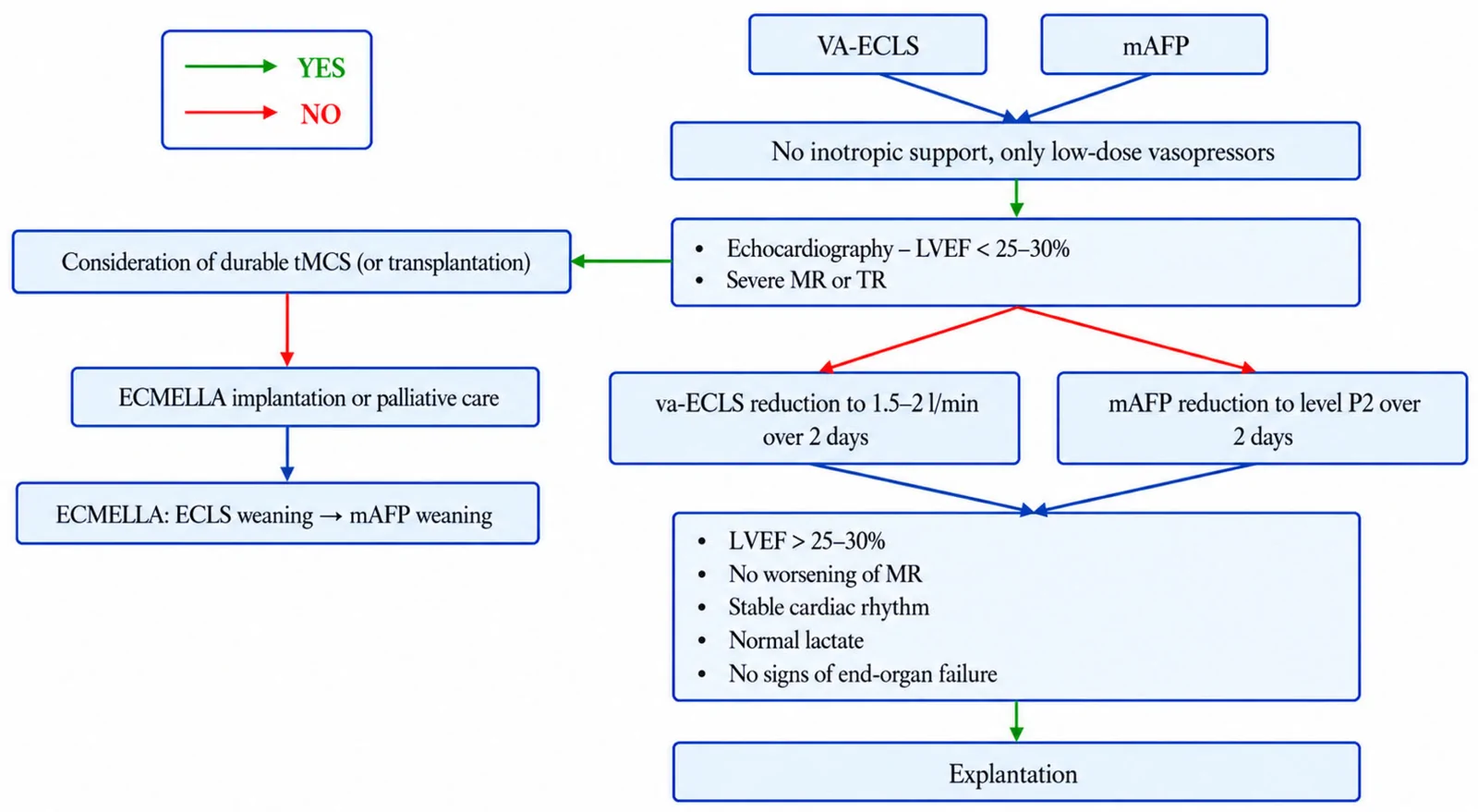
Algorithm for weaning strategies for microaxial flow pumps and VA-ECLS. The algorithm summarizes previously published recommendations and the weaning algorithm described in Temporary Mechanical Circulatory Support in Cardiogenic Shock Patients after Cardiac Procedures: Selection Algorithm and Weaning Strategies [10]. Abbreviations: ECMELLA, extracorporeal membrane oxygenation combined with a microaxial flow pump; LVEF, left ventricular ejection fraction; mAFP, microaxial flow pump; MR, mitral regurgitation; RVEF, right ventricular ejection fraction; tMCS, temporary mechanical circulatory support; TR, tricuspid regurgitation; VA-ECLS, veno-arterial extracorporeal life support.

Consideration should not only be given to tMCS implantation but also to the intended bridge strategy and subsequent device weaning. (Figure 5) Weaning criteria include discontinuation of inotropic support, although low-dose vasopressors may be continued, and an LVEF > 30% at the lowest support level [10]. Stable hemodynamics, metabolic parameters, and end-organ perfusion at the lowest level of support should guide tMCS weaning [8]. For patients with an LVEF below 25%, long-term mechanical circulatory support should be considered. In patients with a LVEF of 25–30%, cardiac function should be reassessed after optimization of heart failure therapy [10]. The EACTS/STS/AATS Guidelines on Temporary Mechanical Circulatory Support in Adult Cardiac Surgery emphasize that VA-ECLS should be used for the shortest possible duration and provide a specific weaning algorithm for each tMCS modality [16].

In summary, several sections of this review demonstrated that current evidence remains limited and is largely based on retrospective studies, registry data, and expert consensus. Therefore, prospective studies are needed to better define patient selection, timing of implantation, and device choice [3,10,126].

## 5. Conclusions

Cardiogenic shock and perioperative low cardiac output syndrome continue to contribute substantially to morbidity and mortality after cardiac surgery. Temporary mechanical circulatory support can be used to maintain hemodynamics and end-organ perfusion, promote myocardial recovery, or bridge patients to further treatment.

Protected cardiac surgery refers to the planned use of tMCS in selected high-risk patients undergoing cardiac surgery. Identifying these patients before surgery and implanting tMCS before severe hemodynamic deterioration may reduce the risk of perioperative LCOS. The proposed classification into three types may help to distinguish different clinical scenarios and support device and timing decisions.

The indication for tMCS should be based on the individual risk profile, ventricular function, planned procedure, and expected perioperative support requirements. Device selection and timing of implantation should be discussed by the Heart Team and supported by institutional protocols. Early LV unloading may be beneficial in selected patients, but the potential advantages must be balanced against bleeding, vascular, hemolytic, and device-related complications.

Further prospective and randomized studies are needed to define optimal timing, device strategies, and long-term outcomes. Overall, protected cardiac surgery represents an important step toward a more preventive and structured approach in high-risk cardiac surgery.

## Figures and Tables

**Table 1:** Clinical scenarios are categorized according to the three types of protected cardiac surgery, with device recommendations based on current European and American guidelines and consensus documents.

Clinical Scenario	Suggested tMCS Device	Timing and Therapeutic Intent	United States Guideline/Consensus Status	European/Joint Surgical Guideline Status
**Type 1** Preoperative haemodynamic, metabolic, or respiratory compromise requiring cardiac surgery, including acute CS [3] BTD/BTS [3]	Predominant LV failure: mAFP or alternative LV support [3,16] Biventricular and/or respiratory failure: VA-ECLS [3,16] Isolated respiratory failure with preserved cardiac function VV-ECLS [3] Add LV unloading during VA-ECLS when indicated (mAFP or IABP) [3,16] Individualize device selection [3,16]	Urgent Heart Team/Shock Team assessment without delaying life-saving treatment [3,16] Initiation before irreversible end-organ injury whenever possible [3,16] Predefined definitive Treatment and exit strategy [3,16]	Selected STEMI with severe/refractory CS: mAFP reasonable—Class 2a, LOE B-R [26] ACS with mechanical complications: short-term MCS as bridge-to-surgery is reasonable—Class 2a, LOE B-NR [26] Routine IABP or VA-ECLS in AMI-CS: not recommended—Class 3: No Benefit, LOE B-R [26] No specific recommendation for pre-emptive tMCS in adult cardiac surgery [26,27]	2023 ESC ACS: Short-term MCS may be considered in severe/refractory CS—Class IIb, LOE C [28] Routine IABP in ACS-related CS without mechanical complications: not recommended—Class III, LOE B [28] 2021 ESC HF: Short-term MCS should be considered in CS as BTR, BTD, or BTB—Class IIa, LOE C [29] 2025 EACTS/STS/AATS: Preoperative tMCS may be considered for refractory structural, haemodynamic, respiratory, or metabolic compromise as a bridge to surgery—Class IIb, LOE B [16] 2025 EACTS PCS consensus: Type 1 framework supporting early, individualized, multidisciplinary stabilization [3] 2026 ESC HF: Selected STEMI-CS with LV dysfunction and no hypoxic brain-injury risk: mAFP should be considered—Class IIa, LOE B1 [24] MI-related mechanical complications: tMCS as bridge to definitive treatment—Class IIa, LOE C [24] Selected patients with HF-related haemodynamic instability: tMCS as BTR/BTD/BTB/BTC/BTT—Class IIa, LOE C [24] Unselected patients with AMI-CS: tMCS not recommended; unselected patients with CS: IABP not recommended—Class III, LOE B1 [24]
**Type 2** Planned intraoperative pre-emptive tMCS [3] Selected patients at high risk of peri-/postoperative LCOS [3,16]	Predominant LV dysfunction: mAFP [3,16] Modest support requirement: selected IABP [3] Predominant RV dysfunction: tRVAD ± oxygenator [3,16] Biventricular/cardiopulmonary dysfunction: VA-ECLS ± mAFP for LV unloading [3,16]	Planned before/at CPB separation [3,16] BTR [3,16] Predefined treatment, weaning and exit strategy [3,16]	No specific contemporary ACC/AHA recommendation [26,27]	2025 EACTS/STS/AATS: intraoperative tMCS should be considered in patients at high risk of post-procedural LCOS to facilitate CPB weaning and BTR—Class IIa, LOE C [16] EACTS PCS consensus: expert consensus; no formal COR/LOE [3] 2026 ESC HF: No specific recommendation for intraoperative tMCS in elective cardiac surgery; recommendations concern selected CS patients [24]
**Type 3** unplanned intraoperative support [3] Failure/difficulty to wean from CPB [3,16]	Correct reversible surgical/technical, rhythm/ischaemic, haemodynamic, respiratory, metabolic and haemostatic causes [3,16] Isolated LV failure: full support mAFP preferred when feasible [16] Biventricular failure: VA-ECLS + LV unloading (mAFP/IABP) or alternative biventricular tMCS [16] Cardiopulmonary failure: VA-ECLS or tLVAD + OxyRVAD [16] Isolated RV failure: tRVAD; add oxygenator if respiratory support is required [3,16] VA-ECLS: consider timely LV unloading for LV distension, absent aortic-valve opening or pulmonary congestion [16]	Immediate tMCS if CPB weaning fails despite optimal measures [16] Avoid delayed escalation and irreversible end-organ injury [3,16]	2022 AHA/ACC/HFSA: General CS recommendation only—tMCS is reasonable when pharmacological therapy cannot maintain end-organ function (Class 2a, LOE B-NR); not CPB-specific [27]	2025 EACTS/STS/AATS: Immediate tMCS is recommended in eligible patients unable to wean from CPB despite optimal measures—Class I, LOE B [16] 2026 ESC HF: Selected HF-related haemodynamic instability: tMCS as BTR, BTD, BTB, BTC, or BTT—Class IIa, LOE C [24] Failure to wean from CPB: no specific recommendation [24]

Abbreviations: AATS, American Association for Thoracic Surgery; ACC, American College of Cardiology; ACS, acute coronary syndrome; AHA, American Heart Association; AMI, acute myocardial infarction; AMI-CS, acute myocardial infarction-related cardiogenic shock; BTB, bridge-to-bridge; BTD, bridge-to-decision; BTR, bridge-to-recovery; BTS, bridge-to-surgery; BTC, bridge to candidacy; BTT, bridge to transplantation; COR, class of recommendation; CPB, cardiopulmonary bypass; CS, cardiogenic shock; EACTS, European Association for Cardio-Thoracic Surgery; ESC, European Society of Cardiology; HF, heart failure; HFSA, Heart Failure Society of America; IABP, intra-aortic balloon pump; LCOS, low cardiac output syndrome; LOE, level of evidence; LV, left ventricle/left ventricular; MCS, mechanical circulatory support; mAFP, microaxial flow pump; OxyRVAD, right ventricular assist device with an oxygenator; PCS, protected cardiac surgery; RV, right ventricle/right ventricular; STEMI, ST-segment elevation myocardial infarction; STS, Society of Thoracic Surgeons; tLVAD, temporary left ventricular assist device; tMCS, temporary mechanical circulatory support; tRVAD, temporary right ventricular assist device; VA-ECLS, veno-arterial extracorporeal life support; VV-ECLS, veno-venous extracorporeal life support.

## Data Availability

No new data were created or analyzed in this review. Data sharing is not applicable to this article.

## Abbreviations

The following abbreviations are used in this manuscript:

tMCS	temporary mechanical circulatory support
mAFP	Microaxial flow pump
LCOS	low cardiac output syndrome
IABP	intra-aortic balloon pump
ECLS	extracorporeal life support
VA-ECLS	veno-arterial extracorporeal life support
LV	left ventricle
EACTS	European Association for Cardio-Thoracic Surgery
CS	Cardiogenic shock
CPB	cardiopulmonary bypass
AMI	acute myocardial infarction
VSD	ventricular septal defect
MR	mitral regurgitation
CABG	coronary artery bypass grafting
OPCAB	Off-pump coronary artery bypass grafting
AATS	American Association for Thoracic Surgery
PCI	Percutaneous coronary intervention
PMR	papillary muscle rupture
TEER	transcatheter edge-to-edge repair
STS	Society of Thoracic Surgeons
LVEDP	left ventricular end-diastolic pressure
LVAD	left ventricular assist device
RVAD	right ventricular assist device
UNOS	United Network for Organ Sharing
